# Adding a treatment arm to an ongoing clinical trial: a review of methodology and practice

**DOI:** 10.1186/s13063-015-0697-y

**Published:** 2015-04-22

**Authors:** Dena R Cohen, Susan Todd, Walter M Gregory, Julia M Brown

**Affiliations:** Leeds Institute of Clinical Trials Research, University of Leeds, Leeds, LS2 9JT UK; Department of Mathematics and Statistics, University of Reading, Reading, RG6 6AX UK

**Keywords:** Adding a treatment arm, Flexible design, Multi-arm multi-stage, MAMS, Adaptive design, Type I error, Family-wise error rate, Statistical methodology, Confirmatory randomised controlled trial, Novel design

## Abstract

Incorporating an emerging therapy as a new randomisation arm in a clinical trial that is open to recruitment would be desirable to researchers, regulators and patients to ensure that the trial remains current, new treatments are evaluated as quickly as possible, and the time and cost for determining optimal therapies is minimised. It may take many years to run a clinical trial from concept to reporting within a rapidly changing drug development environment; hence, in order for trials to be most useful to inform policy and practice, it is advantageous for them to be able to adapt to emerging therapeutic developments. This paper reports a comprehensive literature review on methodologies for, and practical examples of, amending an ongoing clinical trial by adding a new treatment arm. Relevant methodological literature describing statistical considerations required when making this specific type of amendment is identified, and the key statistical concepts when planning the addition of a new treatment arm are extracted, assessed and summarised. For completeness, this includes an assessment of statistical recommendations within general adaptive design guidance documents. Examples of confirmatory ongoing trials designed within the frequentist framework that have added an arm in practice are reported; and the details of the amendment are reviewed. An assessment is made as to how well the relevant statistical considerations were addressed in practice, and the related implications. The literature review confirmed that there is currently no clear methodological guidance on this topic, but that guidance would be advantageous to help this efficient design amendment to be used more frequently and appropriately in practice. Eight confirmatory trials were identified to have added a treatment arm, suggesting that trials can benefit from this amendment and that it can be practically feasible; however, the trials were not always able to address the key statistical considerations, often leading to uninterpretable or invalid outcomes. If the statistical concepts identified within this review are considered and addressed during the design of a trial amendment, it is possible to effectively assess a new treatment arm within an ongoing trial without compromising the original trial outcomes.

## Background

Confirmatory clinical trials can take many years to run, requiring considerable resources. During this time, evidence for a new promising treatment may emerge. It may be advantageous to incorporate the emerging treatment into the ongoing trial as a new randomisation arm. This could be done to ensure that the outcomes of trials are relevant at the time of reporting, whilst benefitting patients, funders, trialists and regulatory bodies by shortening the overall process of comparing and selecting experimental treatments; allowing optimal therapies to be determined faster than would otherwise be the case; and reducing costs and patient numbers. In addition, increasing the number of experimental arms increases the probability of a successful treatment [1].

Ongoing treatment advances are continually improving survival rates in many therapeutic areas, including, for example, most types of cancer [2]. The Cancer Research UK (CR-UK) website states that ‘50% survive 10 or more years’ in UK cancer patients. Improving survival times are fantastic for patients, but increase challenges to researchers in continuing to progress and further improve these survival rates within feasible trials settings. New promising treatments are continually being developed and tested in early phase trials, and it is difficult for researchers to address them in confirmatory trials in a timely manner. It is not appropriate to wait for the results of every promising early phase trial in order to design the next large phase III trial since this would delay the research for the currently available treatments. The ability to add new arms to ongoing trials could help to advance the pace of research by allowing emerging therapies to be investigated in populations where trials already exist without introducing competition and by reducing the set-up time for designing a new trial.

**Figure 1:**
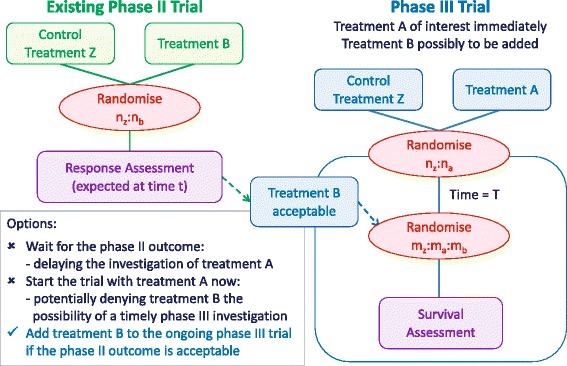
Scenario in which it would be beneficial to add a treatment arm. Illustration of a scenario in which it would be useful to add a treatment arm to a phase III trial. Treatment A is to be assessed in a large phase III trial, but the results of the phase II trial assessing treatment B in the same population have not yet been reported.

The example in Figure 1 illustrates a recent scenario where Treatment A was immediately available for assessment in a large, confirmatory phase III trial in newly diagnosed chronic lymphocytic leukaemia (CLL) patients in the UK. However, a promising Treatment B was undergoing assessment in a phase II trial in the same population against the same standard control group. The phase II trial was shortly due to complete recruitment, but required 12 months of follow-up for the outcomes. The choice was either to delay the assessment of treatment A, therefore denying patients that promising new therapy in a trial setting and delaying the research, or opening the phase III trial and denying treatment B the possibility of a timely phase III investigation in that population. Ideally, the phase III trial assessing Treatment A would be opened now, with Treatment B incorporated at a later time if the phase II evidence was promising.

This review investigates the addition of a new treatment arm to an ongoing trial within the following scope: the trial has already begun recruitment and the randomisation is still open when the new treatment is added, the trial has a confirmatory primary objective, the trial is designed using frequentist methodology (due to the differences in assumptions and considerations with Bayesian methodology), and the entire treatment arm is new rather than an amendment to an existing arm.

An initial literature review found only a small number of publications that mentioned the concept of adding an arm, and there was no comprehensive research or guidance on how to do this whilst maintaining the statistical integrity of the trial. If the amendment compromises the statistical validity of the trial such that a primary hypothesis cannot be answered, it may render the trial unethical and waste resources.

The aims of this manuscript are to summarise the current literature regarding statistical methods and design considerations when adapting a trial by adding a new treatment midway through recruitment and to investigate trials that have added an arm in practice and how they addressed the statistical considerations.

## Review

### Methods

A comprehensive literature review was conducted to obtain and assess all current literature regarding statistical methods and design considerations when adapting an ongoing trial by adding a new treatment arm. A protocol was written in advance to fully define the aims, methods and search strategy. Search terms were defined for the following major electronic databases: MEDLINE (Ovid), EMBASE (Ovid), Science Citation Index (Web of Science) and the Cochrane Library (Wiley), each from inception. The ProQuest database was also searched to identify further relevant grey (unpublished) material such as dissertations and theses, and conference papers. The search terms are provided in the Appendix. The search was conducted in November 2012, and auto alerts were set up to identify any further literature that arose by the time of publication. In order to identify any additional publications, searches were performed on references, authors and citations of directly relevant literature.

In addition, an assessment of summary, regulatory, guidance and review documents on flexible or adaptive designs in general was undertaken to identify methodologies that have been investigated and published that may be relevant. Many of these were collaborative works from groups of experts. They included a handbook [3], regulatory documents [4-6], and publications from statistical collaborative groups [7,8]. A search was also conducted in MEDLINE to identify any further key overview documents. Titles and abstracts were scanned for direct relevance to general guidance or review documents, but not including documents relating to a particular disease or methodology. The types of adaptation and key statistical considerations discussed in each document were listed and summarised, and their relevance was determined in discussions within the research team.

Both methodological and practical publications on trials that may have implemented this adaptation were deemed relevant, if they were within scope as described in the introduction. However, practical examples of trials that have implemented this research idea by adding a new treatment part way through recruitment were rarely identified using literature searches as the design amendment was not the primary aim of results publications. In order to identify as many trials as possible, key statisticians and researchers were contacted directly, and references from relevant methodological papers were reviewed. Twenty-two statisticians or researchers from UK Clinical Research Collaboration (UKCRC) registered trials units were contacted, along with six prominent international researchers and two large UK funding bodies for cancer trials. In addition, national and international conference, workshop and forum presentations aimed to target wide audiences of trialists in order to gain awareness of further relevant trials and keep abreast of developments in this area.

### Literature on the methodology of adding arms

Only seven publications were identified that discussed any methodological considerations when adding an arm to an ongoing trial. These were reviewed in detail to assess and summarise the research previously carried-out and the recommendations or methodology discussed.

Phillips *et al*. [8] summarise discussion points on adaptive designs from the Statisticians in the Pharmaceutical Industry (PSI) Adaptive Design Expert Group. There is a brief paragraph stating that it is possible to add new treatment arms, although no details or relevant considerations are provided. Three references are noted, one is methodological [9], one is out of scope as it is based on exploratory dose finding endpoints [10], and the other is a practical results paper [11].

Elm *et al*. [12] provide a very relevant paper on ‘flexible analytical methods for adding a treatment arm mid-study to an ongoing clinical trial’, which considers adding an independent treatment based on external considerations. The main aim is to compare methods for analysing continuous, normally distributed, outcome data over the stages (before and after the amendment), accounting for potential differences in patient cohorts. The analysis adjusts the allocation ratio so that all three arms complete recruitment at the same time with the same patient numbers, although that leads to a reduced power for the new comparison due to the lower numbers in the concurrent placebo control group. Three analysis methods were compared using simulations, with varying assumptions around the intra-stage correlation caused by the addition of the new treatment. These methods were to simply pool the data over the stages, to apply a linear model adjusting for the design change, and to use adaptive methodology to calculate *P* values separately for each stage and combine them using combination test principles [13]. The results showed that when there is a correlation, there is bias and a loss of power for both comparisons when the data are simply pooled, but particularly for the new comparison as would be expected due to the use of non-concurrent control placebo data. In the reported scenario, the linear model was the most powerful since there is a loss of power associated with the use of closed testing procedures, but a combination test was thought better if there are amendments to the original trial alongside the addition of the arm.

Sydes *et al*. [14] discuss ‘STAMPEDE’, an ongoing multi-arm multi-stage (MAMS) randomised, controlled trial, designed to be able to drop and add arms throughout the recruitment period. The publication is not written as a general guidance document, but presents trial-specific methodological and practical issues. At the time of publishing, a new research arm had been added to the existing control and five experimental arms, based on the parameters and targets designed at the outset. The trial has a pragmatic design where only concurrent controls are used for comparison with the new treatment, and since the experimental arms are not formally compared against each other, no type I error adjustment is made for multiplicity.

Wason *et al*. [15] make general recommendations for MAMS trials, including a section on adding treatment arms at planned interim analyses. The example is theoretical, based on continuous outcomes, and focuses on strong family-wise error rate (FWER) control due to multiple arms, that is, the probability of making any type I errors over the trial as a whole. The discussion argues against the use of pairwise error rates (PWER), the probability of making a type I error within individual comparisons within a trial, because this situation is ‘conceptually quite different to running a series of separate trials’ and strong FWER control is required for confirmatory claims.

Hommel [9], Posch *et al*. [16] and Bauer *et al*. [17] mention adding an arm or hypothesis as being possible within a flexible framework, although the primary purpose of the papers are to discuss methodology for controlling FWERs when various types of design adaptation are made, usually at internal interim analyses. Methods are based on adaptive combination test principles to analyse the data by stage. Posch acknowledges that the test is stringent in controlling alpha, but may give a large penalty in terms of power, saying ‘This is the price to be paid for the great flexibility provided by the adaptive design’.

In addition, two text books were identified with chapters that made a reference to adding an arm to an ongoing trial [3,18]. The chapters were contributed by Hommel and Posch respectively, and contain similar ideas to the publications discussed above.

### Literature on adaptive designs in general

The assessment of summary, regulatory, guidance and review documents on flexible or adaptive designs in general was undertaken as described in the methods section. In addition to the documents previously referenced [3-8], a further 13 were identified for detailed review from the MEDLINE search [19-31]. None of these documents discussed the addition of a trial arm. The statistical considerations discussed within these documents were summarised and assessed for relevance to this situation, and those considerations identified to have relevance are included within the summary below.

### Key statistical considerations when adding a treatment arm to an ongoing trial

**Figure 2:**
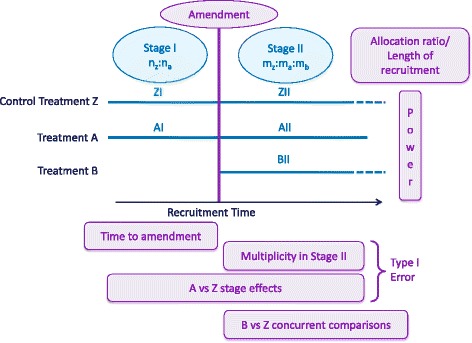
Trial timeline in which an arm is added, highlighting the key statistical considerations. Illustration of a trial timeline in which an arm is added as an amendment. The trial has two distinct stages, and the key statistical considerations are displayed.

This literature review on methodology when adding arms and on adaptive designs in general generated the identification of a number of statistical considerations with relevance when amending ongoing clinical trials by adding a new treatment arm based on external evidence. The main considerations identified are illustrated in Figure 2, and summarised here. This section is intended to aid the implementation and review of this type of design amendment by summarising the main statistical issues that need to be addressed, and the related views within the literature. Each of these should be considered on a trial by trial basis during the planning stages.

#### Controlling error due to stage effects

The primary statistical issue in most methodological publications discussing adaptive or flexible designs is strong control of the FWER, the probability of making at least one type I error over the trial as a whole. Of the seven relevant methodological publications, all but one (STAMPEDE [14]) focus primarily on closed-testing analysis methods, in which analysis is performed by stage, and a combination function is used to derive an overall test statistic, conserving the FWER. Typically in adaptive designs, the amendment is based on interim data that are internal to the trial being adapted, so it is necessary to analyse by stage and use *P* value combination methods in order to control the overall FWER in the strong sense [13]. In this case, however, it is assumed that the arm is added based on external information with no looks at internal trial data required, and therefore the FWER would not be inflated due to interim analyses.

When data are analysed over both stages combined, there might be a stage effect bias due to the treatment effects differing over the stages, possibly due to a shift in patient population at the time of the amendment. This shift could be caused by, for example, potential toxicity, promising early efficacy data or a change in eligibility criteria. A different treatment effect in each treatment group could cause a treatment*stage interaction. Simply pooling the data over stages for analysis could lead to a biased outcome, and therefore, it may be necessary to analyse the stages separately.

It was noted, however, that *P* value combination techniques, whilst strongly controlling the FWER, may have a large penalty in terms of power [16]. The power to detect a treatment*stage interaction is likely to be small; however, if there is no indication that an interaction exists in terms of statistical significance, no clinical justification for any stage effects and no changes to eligibility, then using *P* value combination methodology might be inappropriately conservative. Elm [12] investigated the use of a multivariate model to adjust for stage as an alternative strategy, although this has not been widely discussed in the literature. They found this approach to be more powerful compared to combination methods.

Assuming no interim data has informed the amendment, *P* value combination methods may be overly stringent, but a stage effect and treatment*stage interaction should be accounted for within a multivariate analysis approach to prevent bias.

Referring to Figure 2, stage effects would only affect the comparison for the original experimental arm (A versus Z), since the new arm does not exist in the first stage.

#### Family-wise error rate control due to multiplicity

When a third arm is added to a trial, multiplicity concerns are introduced due to multiple primary comparisons within the same trial based on a shared control arm. There are conflicting views within the literature on whether strong control of the FWER is needed in this case, or whether it is adequate to control the PWER for each experimental arm versus control. Assuming the experimental arms are independent, the primary analyses are restricted to comparisons against control rather than pairwise comparisons, the arms are being tested in the same trial for only efficiency purposes, and the amendment is not based on internal interim data, it has been argued that this is analogous to running separate trials and therefore that FWER control is not necessary [14,32]. However, others argue that multiplicity issues arise due to multiple use of the same control population and that strong FWER control is a regulatory requirement for confirmatory claims [4,15,33]. Regulators [33,34] advocate that strong FWER control is required in cases where there are more than two treatment arms, which is likely to be the safer choice for making confirmatory claims as long as the power remains adequate. The level of control needs to be carefully considered and justified on a trial-by-trial basis.

#### Non-concurrent control data

If there is a shift in the patient population in the second stage, after the new arm has been added, the control data collected prior to the amendment may have a different survival pattern to that collected after. For this reason, the control data collected prior to the amendment may not be an unbiased comparator for the new arm. One of the methodological papers stipulates the use of concurrent controls [14]. The others do not discuss this directly, but by advocating methods for analysing the data by stage and then combining the p-values, this is implicit.

#### Power recalculation

When a new treatment is included within a confirmatory trial, care needs to be taken that there is adequate power to assess the primary hypothesis associated with that treatment. In addition, the literature recognises that strong control of the FWER will reduce the power [18], which should be accounted for to ensure that the power remains adequate for the existing and new hypotheses.

#### Determinants of efficiency

The allocation ratio and length of recruitment to each treatment arm could be adjusted to improve efficiency in terms of total number of patients required and time taken to answer the primary hypotheses, and need to be carefully balanced considering the requirements for the trial in addition to the viewpoints within the literature described here.

Dunnett [35] showed that the optimal allocation to the control group in multi-arm trials is approximately the square root of the number of experimental arms in order to minimise the total numbers of patients required. Wason *et al*. [15] investigated the optimal allocation ratio in MAMS trials with varying numbers of experimental arms and numbers of stages, allowing for early stopping. They found that ‘Although efficiency (in terms of maximum sample size) can be gained by deviating from an optimal allocation to each arm, the gain is generally fairly small’. This was due to the chance of experimental arms being dropped at each stage, suggesting that optimal allocation is not necessarily straightforward where the number of treatment arms varies throughout the trial. Patient acceptability also needs to be considered, since the more attractive a trial (often perceived as being related to the higher the chance of receiving an experimental treatment) the better recruitment rates tend to be.

Elm *et al*. [12] believe that the allocation ratio should be adjusted so that all arms complete recruitment at the same time to ensure maintenance of blinding and to prevent a ‘stage III’ effect due to dropping the original arm. Other trialists such as those who design MAMS trials, however, advocate that arms can be added or dropped throughout the trial at different stages as required, which leads to a rolling design where outcomes become available for analysis at different times within different arms.

#### Changes to the control group

Potentially, a new therapy may receive approval, which would make the existing control group inferior to standard of care and, therefore, unethical. No methodological papers mention changing the control group, but for long or rolling trials this is something that is likely to arise. This could cause complex issues, and needs careful consideration.

#### Logistical considerations

There are many important logistical considerations discussed within the literature that need to be overcome for the amendment to be feasible. They include: funding; time taken to implement the amendment; approvals; acceptability to funders, patients, regulators and researchers; blinding; changes to data management systems; interaction with and inclusion of the Data Monitoring Committee (DMC) and Trial Steering Committee (TSC); centre approvals; recruitment strategy, whether to pause or continue; and feasibility of including treatments produced by different pharmaceutical companies.

### Practical examples of ongoing trials in which an arm has been added

A search of the literature only identified three examples of trials in which an arm had been added, two results papers and the Sydes *et al*. publication, which also includes the methodological discussion [14]. However, the direct contacts described in the Methods section suggested 30 unique trials. Eight trials were identified to be within scope. All of these trials had obtained appropriate ethical approval.

Two trials have MAMS designs and six are large phase III trials published in high-impact journals, some of which went on to change clinical practice.

AML16 [36] and STAMPEDE [14,37,38] have MAMS designs, each having added new arms, based on predetermined criteria. One arm was added to the non-intensive randomisation for AML16 when there were three arms already in the trial, 4 years into the 5-year recruitment period. Publications report some pairwise comparisons arising from this trial, but the outcomes for the new arm have yet to be published. STAMPEDE is still open to recruitment and has currently added three arms 6, 7 and more than 8 years after the trial opened. Some of the original arms had closed to recruitment when the arms were added, but the control arm remains open. All arms are still in follow-up for their primary outcome measure.

The 2NN trial [11] was a large international HIV trial (Lancet, 2004). The new arm became the control, and the overall numbers were not increased thus reducing the power for all comparisons. The authors refer to the addition as a drawback and state that the overall efficacy estimates should be interpreted with caution, but believe that the main conclusions of the study are robust.

CATIE [39] was a double-blind schizophrenia trial (NEJM, 2005). A fifth arm was added after 1 year of recruitment following FDA approval. Patient numbers were not increased to the trial as a whole, and an even allocation ratio led to approximately 50% power for the new comparison. The trial statistician said they had ‘a limited budget and could not add enough patients for good power, yet it was felt by investigators that if it was not added, then the study might be missing an important evaluation’.

SANAD [40] was an Health Technology Assessment (HTA) funded epilepsy trial, (Lancet, 2007). An unplanned fifth arm was added within a non-inferiority setting but without an increase to the trial size, leading to the conclusion for the new arm that ‘the smaller numbers of patients available to the comparison reduce the statistical power and we could not conclude that they are equivalent’.

AML15 [41,42] was a complex trial where a new arm was added to the induction randomisation, which had a 2 x 2 factorial design. The new treatment was originally added alone, but later was amended to include the factorial randomisation to make it a 3 x 2 design. The results or statistical considerations when adding the arm have not been published.

N9741 [43,44] and N0147 [45] were large, practice-changing, US regulatory colorectal cancer trials (JCO 2004, JAMA 2012). During the trials, there were a number of treatments added and dropped, although care was taken to control the power. The final publication included only the arms that remained after the amendments had taken place.

### Statistical considerations in practice

The statistical considerations that were identified within the methodological literature have been addressed to varying extents in practice. Note that not all considerations need to be addressed in each case because of the nature of the trials and their objectives. When designing or critically evaluating the results of different trials, it should be determined for each trial whether the conditions are necessary for the results to be robust, or advantageous to improve efficiency or feasibility.

None of the trials analysed the results by stage, and only one (STAMPEDE [14]) reported adjusting for stage or treatment*stage interaction within multivariate analyses.

Of the trials that added an arm, four strongly controlled the family-wise error rate for multiplicity due to having more than one primary comparison, and four did not, of which two were MAMS trials that primarily compared each experimental arm only to control.

Seven of the eight trials used concurrently recruited control patients only. The other (2NN [11]) tested for an intra-stage correlation before pooling the data, although power for this test to detect a treatment*stage interaction was likely to be low.

Only five of the eight trials controlled the power for the existing and new hypotheses. The others were underpowered for some or all primary comparisons due to the amendment, and all reported this as a limitation. No trials adjusted the power to account for FWER control.

Only two trials deviated from a 1:1 allocation ratio: 2NN [11] changed from its original 1:1:1 design to recruit at 1:2:2:1 after the amendment for practical reasons, and STAMPEDE [14] recruited more to control initially because ‘It is more efficient to have more patients allocated to the control arm when there are more research arms co-recruiting’. Once some arms had been dropped and there were fewer experimental arms, the new comparisons were randomised with even allocation, although the allocation ratios remained constant within any given comparison. All but STAMPEDE [14] stopped recruitment at the same time in all confirmatory arms, excluding those that were dropped early at interim analyses.

Although none of the methodological papers mentioned changing the control group, two trials have done so when adding a new arm in practice. The 2NN trial [11] amended the primary hypotheses so the new arm became the control group for all primary analyses. N9741 [43] changed the control group for the whole trial to one of the existing experimental arms because of a change in the standard of care, requiring the original control arm to be dropped.

## Conclusions

Recent initiatives in clinical trials are aimed at speeding up research by making better use of scarce resources. For example, the FDA’s Critical Path Initiative ‘to drive innovation in the scientific processes through which medical products are developed, evaluated, and manufactured’ included the production of guidance on adaptive designs to increase the efficiency of studies. In the UK, the National Institute for Health Research Health Technology Assessment (NIHR HTA) programme recently released a call for ‘Efficient Study Designs’ with a focus on research that ‘will demonstrate particular design features to allow either more rapid conduct, or lower costs’. It is clear that there is the demand to improve the efficiency of clinical trials in order to speed up the overall process of getting the best therapies to patients. If a suitable treatment emerges whilst a trial in a similar population is ongoing, there would be many advantages to modifying the existing trial by adding the new arm, as long as the statistical considerations are addressed appropriately.

This literature review has confirmed that very few publications have addressed the topic of how to add a treatment arm to an ongoing trial, and none have done so either systematically or comprehensively.

Only a very small number of trials were identified to have added arms in practice, indicating that although this type of amendment may be advantageous, it is very rarely implemented. Of the trials that had added an arm, some failed to adequately address the statistical issues and suffered from lack of power and difficulties of interpretability. However, it is clear that this type of amendment is desirable and advantageous, with the statistical and logistical issues seeming by no means insurmountable.

Guidance is needed to allow amendments that add new arms to existing trials to be made only with robust statistical integrity. The benefits in cost, time and patient resource savings from such amendments are clearly very substantial; and therefore further methodological work in this area is the subject of current research, so that the addition of new arms to existing trials can, in the future, be recommended and encouraged.

## Appendix

Literature review search strategies by database

***MEDLINE***

1. Research Design/
2. exp Clinical Trials as Topic/
3. 1 or 2
4. ((adaptive adj3 design*) or (adaptive adj3 method*) or (adaptive adj3 trial*)).mp.
5. ((flexible adj3 design*) or (flexible adj3 method*) or (flexible adj3 trial*)).mp.
6. ((multi?stage adj3 design) or (multi?stage adj3 method*) or (multi?stage adj3 trial*)).mp.
7. 4 or 5 or 6
8. ((adding or additional or incorporat* or extra) adj4 (arm* or treatment* or group* or therap* or randomi* or hypothes*)).mp.
9. 3 and 7 and 8

***EMBASE***

1. Methodology/
2. exp “Clinical Trial (Topic)”/
3. 1 or 2
4. ((adaptive adj3 design*) or (adaptive adj3 method*) or (adaptive adj3 trial*)).mp.
5. ((flexible adj3 design*) or (flexible adj3 method*) or (flexible adj3 trial*)).mp.
6. ((multi?stage adj3 design) or (multi?stage adj3 method*) or (multi?stage adj3 trial*)).mp.
7. 4 or 5 or 6
8. ((adding or additional or incorporat* or extra) adj4 (arm* or treatment* or group* or therap* or randomi* or hypothes*)).mp.
9. 3 and 7 and 8

***Web of Knowledge (Web of Science)***

Topic = (adaptive near/3 design* or adaptive near/3 method* or adaptive near/3 trial* or flexible near/3 design* or flexible near/3 method* or flexible near/3 trial* or multi$stage near/3 design* or multi$stage near/3 method* or multi$stage near/3 trial*) AND Topic = ((adding or additional or incorporat* or extra) near/4 (arm* or treatment* or group* or therap* or randomi* or hypothes*))

***Cochrane Library***

1. MeSH descriptor: [Research Design] explode all trees
2. MeSH descriptor: [Clinical Trials as Topic] explode all trees
3. 1 or 2
4. (adaptive near/3 design* or adaptive near/3 method* or adaptive near/3 trial* or flexible near/3 design* or flexible near/3 method* or flexible near/3 trial* or multi?stage near/3 design* or multi?stage near/3 method* or multi?stage near/3 trial*):ti,ab,kw and (adding or additional or incorporat* or extra) near/4 (arm* or treatment* or group* or therap* or randomi* or hypothes*):ti,ab,kw (Word variations have been searched)
5. 3 and 4

***Proquest Scholarly Journals***

(adaptive NEAR/3 design* OR adaptive NEAR/3 method* OR adaptive NEAR/3 trial* OR flexible NEAR/3 design* OR flexible NEAR/3 method* OR flexible NEAR/3 trial* OR multi*stage NEAR/3 design* OR multi*stage NEAR/3 method* OR multi*stage NEAR/3 trial*

AND

all ((adding OR additional OR incorporat* OR extra) NEAR/4 (arm* OR treatment* OR group* OR therap* OR randomi* OR hypothes*))

AND

su. Exact (“research design (72950)” OR “research design” OR “clinical trials, phase ii as topic” OR “research design (04701)” OR “clinical trials, phase iii as topic” OR “clinical trials as topic” OR “clinical trials” OR “clinical trials data monitoring committees”)

Additional limits - Source type: “Conference Papers & Proceedings“ OR ”Scholarly Journals” OR “Dissertations & Theses")

## Abbreviations

CLL: chronic lymphocytic leukaemia, a cancer of the white blood cells
CR-UK: Cancer Research UK
DMC: data monitoring committee
EMEA: European Medicines Agency
FDA: US Food and Drug Administration
FWER: family-wise error rate
HTA: NIHR Health Technology Assessment
MAMS: multi-arm multi-stage
NHS: National Health Service
NIHR: National Institute for Health Research
PhRMA: Pharmaceutical Research and Manufacturers of America
PSI: Statisticians in the Pharmaceutical Industry
PWER: pairwise error rate
TSC: trial steering committee
UKCRC: UK Clinical Research Collaboration
